# Improved joint X-ray and neutron refinement procedure in *Phenix*

**DOI:** 10.1107/S2059798323008914

**Published:** 2023-11-09

**Authors:** Dorothee Liebschner, Pavel V. Afonine, Billy K. Poon, Nigel W. Moriarty, Paul D. Adams

**Affiliations:** a Molecular Biosciences and Integrated Bioimaging Division, Lawrence Berkeley National Laboratory, Berkeley, CA 94720, USA; b Department of Bioengineering, University of California, Berkeley, Berkeley, CA 94720, USA; MRC Laboratory of Molecular Biology, United Kingdom

**Keywords:** macromolecular crystallography, neutron diffraction, joint XN refinement

## Abstract

Neutron diffraction is one of the three crystallographic techniques (X-ray, neutron and electron diffraction) used to determine the atomic structures of molecules. Its particular strengths derive from the fact that H (and D) atoms are strong neutron scatterers, meaning that their positions, and thus protonation states, can be derived from crystallographic maps. However, because of technical limitations and experimental obstacles, the quality of neutron diffraction data is typically much poorer (completeness, resolution and signal to noise) than that of X-ray diffraction data for the same sample. Further, refinement is more complex as it usually requires additional parameters to describe the H (and D) atoms. The increase in the number of parameters may be mitigated by using the ‘riding hydrogen’ refinement strategy, in which the positions of H atoms without a rotational degree of freedom are inferred from their neighboring heavy atoms. However, this does not address the issues related to poor data quality. Therefore, neutron structure determination often relies on the presence of an X-ray data set for joint X-ray and neutron (XN) refinement. In this approach, the X-ray data serve to compensate for the deficiencies of the neutron diffraction data by refining one model simultaneously against the X-ray and neutron data sets. To be applicable, it is assumed that both data sets are highly isomorphous, and preferably collected from the same crystals and at the same temperature. However, the approach has a number of limitations that are discussed in this work by comparing four separately re-refined neutron models. To address the limitations, a new method for joint XN refinement is introduced that optimizes two different models against the different data sets. This approach is tested using neutron models and data deposited in the Protein Data Bank. The efficacy of refining models with H atoms as riding or as individual atoms is also investigated.

## Abstract 2

The improved joint X-ray and neutron refinement procedure in *Phenix* optimizes two different models against the X-ray and neutron data sets. This approach is shown to reduce overfitting compared with refining the models separately.

## 1. Introduction

Neutron macromolecular crystallography is a diffraction method (Shull & Wollan, 1948 ▸) that can be used to determine the atomic structures of biomolecules. Neutron diffraction relies on the same fundamental concepts as X-ray diffraction, which is the predominant method of determining the three-dimensional structures of macromolecules. In both methods, major peaks in Fourier maps equate to atomic positions that can be used to determine the structure. Nuances result from the nature of the interaction; while X-rays interact with the electron cloud of atoms, neutrons interact with the atomic nuclei (considered a point scatterer). For X-rays, the scattering length varies linearly with the number of electrons, so that hydrogen (H), which possesses only one valence electron, does not produce enough signal to be observed in electron-density maps (unless the data resolution is very high, typically around 1 Å; Petrova & Podjarny, 2004 ▸). In contrast, the neutron scattering cross section varies by element or isotope in a nonlinear fashion, resulting in the scattering lengths of hydrogen and deuterium (D) atoms being similar to those of heavier atoms (C, O and N). This allows the positions of H and D atoms to be determined from the diffraction data. These properties enable neutron crystallography to provide the protonation states of amino-acid side chains and the orientations of some water molecules. This information is often crucial for understanding the reaction pathways of proteins (Kono & Tamada, 2021 ▸).

**Figure 1:**
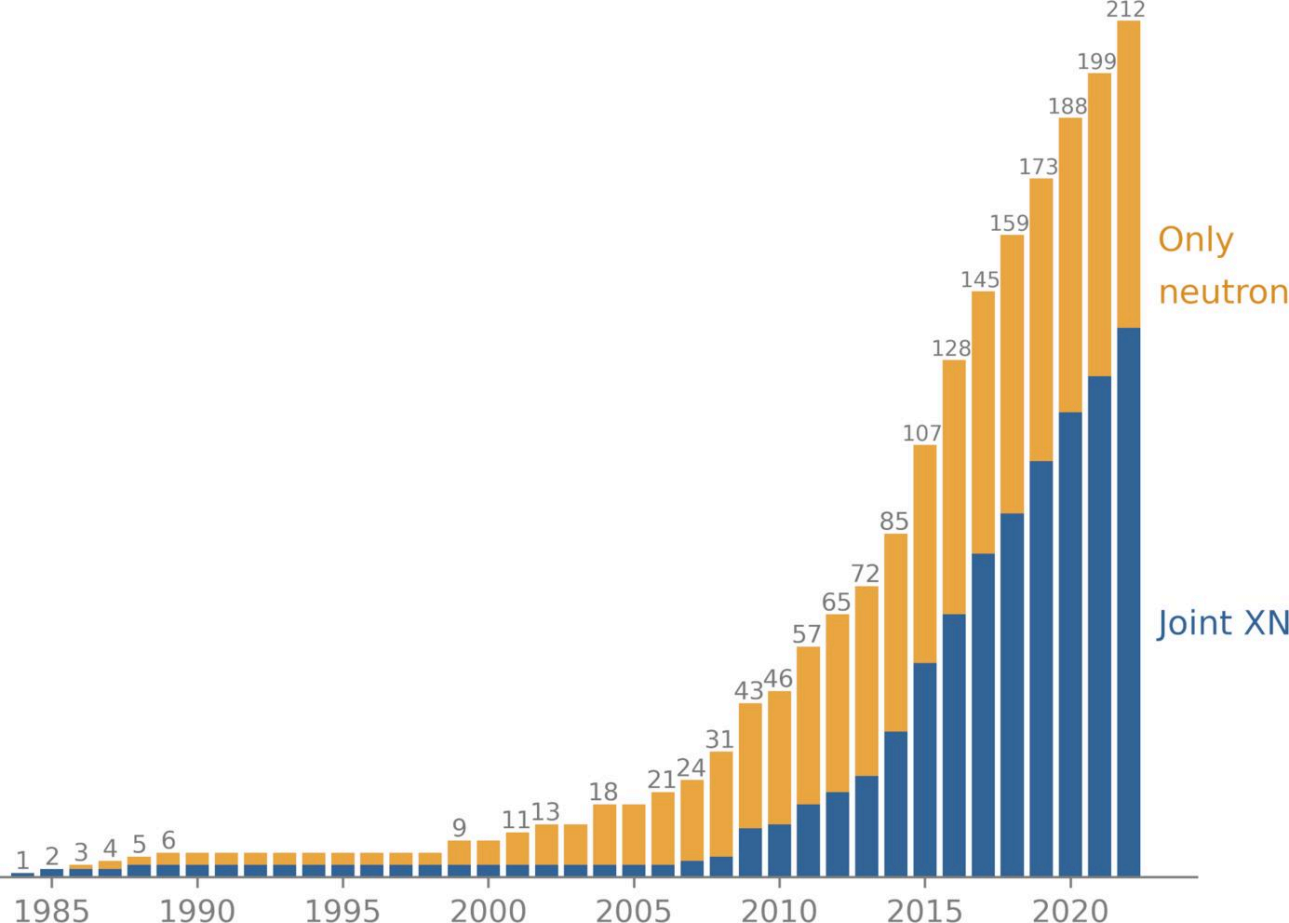
Cumulative number of neutron model depositions in the PDB per year (based on the PDB deposition date).

**Figure 2:**
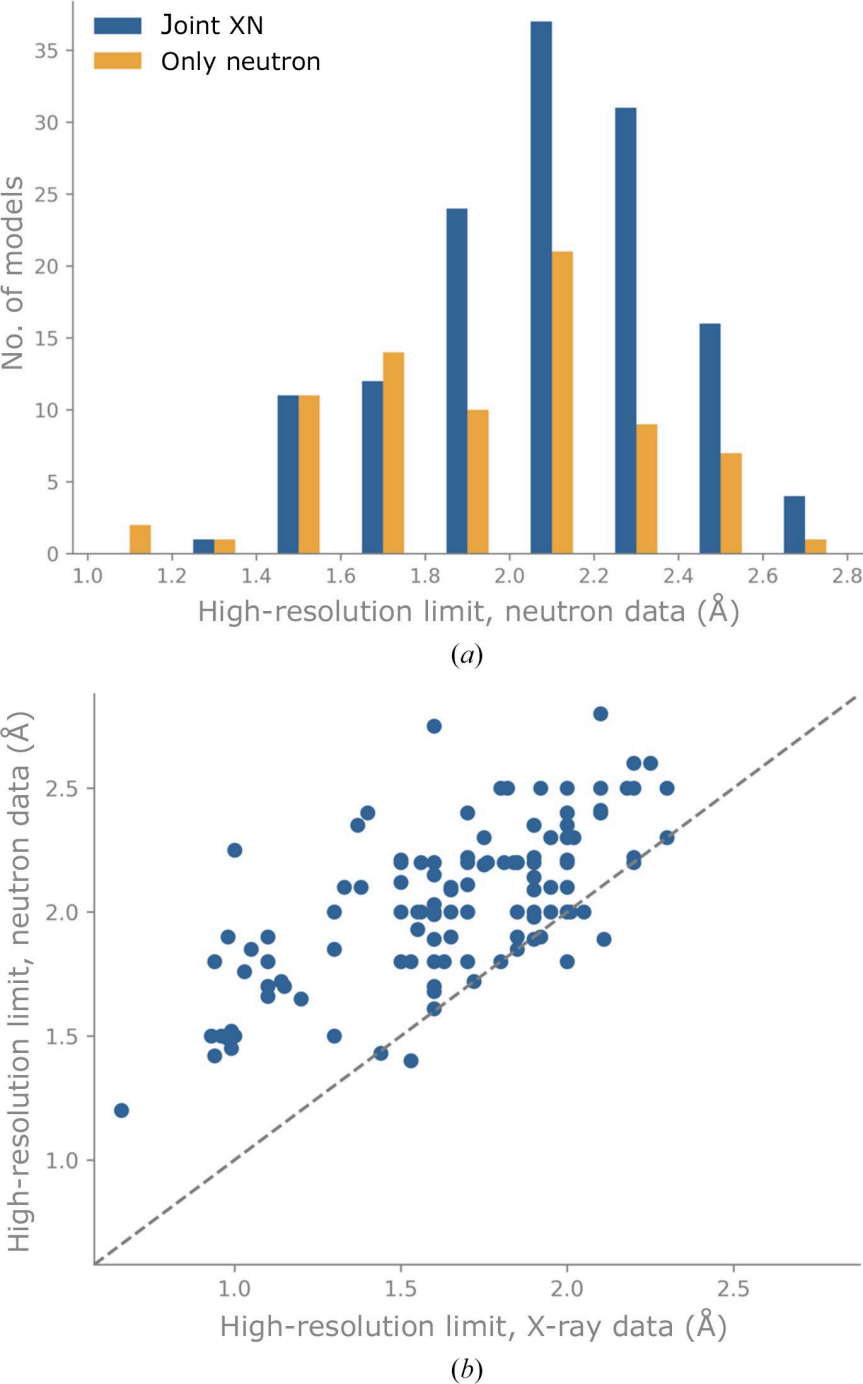
(*a*) Histogram of the high-resolution limit for neutron data (retrieved from PDB file header or from primary citations). Bin width 0.2 Å, ticks mark bin limits. (*b*) High-resolution limit *d*_min_ of the neutron data against that of the concomitant X-ray data for joint XN entries. The dashed line represents the bisector.

Unfortunately, practical reasons impede the routine application of neutron macromolecular crystallography. As the beam flux at neutron sources is relatively weak, neutron diffraction requires large crystals (typically at least 0.1 mm^3^) and long data-collection times, ranging from several days to several weeks (Howard *et al.*, 2011 ▸; Weber *et al.*, 2013 ▸; Ng *et al.*, 2015 ▸). It can be challenging to obtain large crystals for a sample that is desired to be investigated by neutron macromolecular crystallography, so the number of samples that can be explored using this method is relatively small (Fig. 1 ▸). Furthermore, the number of neutron structures is limited by the small number of beamlines dedicated to neutron macromolecular crystallography. These beamlines are typically over-subscribed, suggesting that many more structures could be obtained if more beamlines were available. Other practical challenges originate from the neutron diffraction properties of hydrogen. As the incoherent scattering cross-section of hydrogen is large, the background level of diffracted beams is high. In addition, the hydrogen scattering length is negative, so that the nuclear scattering length density can cancel out for groups such as CH_2_. In contrast, deuterium has a much smaller incoherent scattering cross section and also a larger coherent scattering cross section, resulting in an increased signal-to-background ratio. Also, the scattering length of deuterium is positive so there is no cancelation effect. It is thus preferable to partially or fully replace hydrogen with deuterium, which can be achieved by soaking the crystal in deuterated buffer solutions or by performing protein expression in fully deuterated reagents, respectively. We note that obtaining fully deuterated (perdeuterated) samples is a costly and time-consuming process (Fernandez-Alonso & Price, 2017 ▸) that can be an experimental obstacle. Also, the data resolution achieved by neutron diffraction is often inferior to that obtained with X-ray diffraction (Fig. 2 ▸).

**Figure 3:**
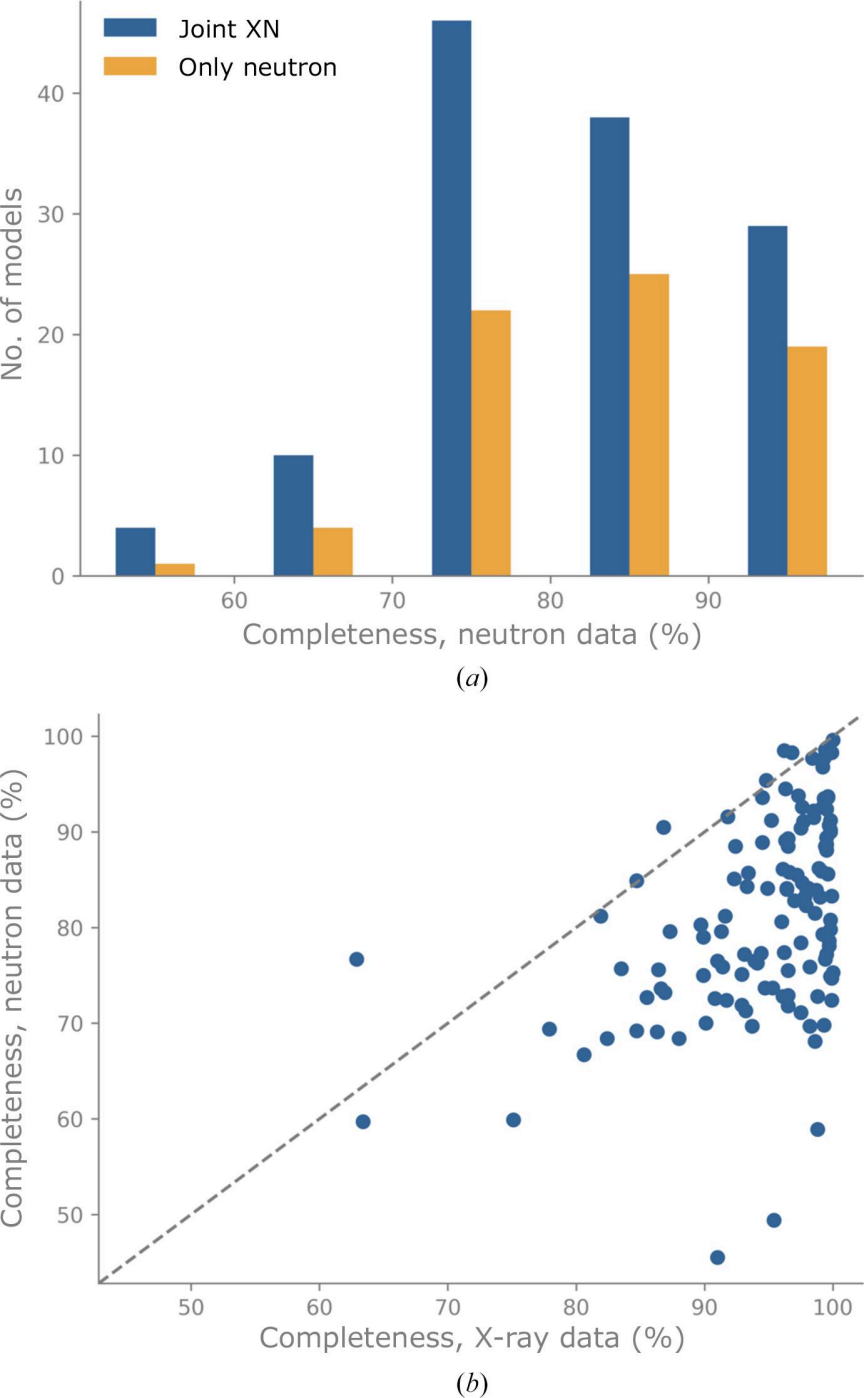
(*a*) Histogram of neutron data completeness calculated from the deposited data. Bin width 10%, ticks mark bin limits. (*b*) Completeness of the neutron data against that of the concomitant X-ray data for joint XN entries. The dashed line represents the bisector.

The experimental challenges (low flux, reduced signal to noise and limited data-collection time) are the reason that the completeness of neutron diffraction data is typically low, reaching approximately 82% on average across all neutron model depositions (Fig. 3 ▸). In comparison, an X-ray data set is considered to be of good quality if the completeness is at least 95% (Dauter & Dauter, 2017 ▸).

As H (or D) atoms are strong scatterers in neutron diffraction, they will appear in nuclear scattering length density maps and thus need to be included in the model and in refinement. The number of parameters to be refined increases substantially as about half of the atoms in a protein are H atoms (Afonine & Adams, 2012 ▸). The number of parameters increases further if the H atoms in the sample have been only partially exchanged with D atoms, as different levels of H/D exchange result in scenarios where there can be both H and D atoms, or either of them, at one location. Such an increase in refinable parameters leads to the risk of data overfitting, which is exacerbated by the typically low completeness, resolution and signal to noise of neutron diffraction data.

In X-ray crystallography, where H atoms are usually not observed (Meents *et al.*, 2009 ▸; Petrova & Podjarny, 2004 ▸), but their inclusion is nonetheless beneficial to refinement, this challenge is addressed by the use of the ‘riding hydrogen’ model (Sheldrick & Schneider, 1997 ▸). In this strategy, the coordinates of the H atoms are inferred from the locations of their covalently bound neighbors. The riding model can, in principle, also be used for refinement against neutron data, but this strategy may obfuscate the information about H/D atoms that is contained in the diffraction data.

Another strategy to address the challenge of refining many parameters while simultaneously dealing with poor data quality is to perform joint X-ray and neutron refinement (hereafter referred to as joint XN refinement; Coppens, 1967 ▸; Orpen *et al.*, 1978 ▸; Wlodawer, 1980 ▸; Wlodawer & Hendrickson, 1982 ▸; Adams *et al.*, 2009 ▸; Afonine *et al.*, 2010 ▸). In joint XN refinement, a single model is simultaneously refined against X-ray and neutron data using a combined refinement target function,

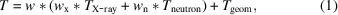

where *T*_X-ray_ and *T*_neutron_ are target functions relating model and X-ray or neutron data, *T*_geom_ is a restraints term that adds *a priori* information and *w*, *w*_x_ and *w*_n_ are weights. The joint XN approach is facilitated by the fact that X-ray data and the corresponding crystal structure models are usually available prior to structure determination with neutron data. Thus, the joint XN refinement method has been widely adopted, accounting for about two thirds of neutron models deposited in the Protein Data Bank (PDB; Berman *et al.*, 2000 ▸; 136 out of 212 neutron models as of April 2023).

Using the refinement target in (1) with a single model assumes that the X-ray and neutron data sets originate from the same crystal. Also, it assumes that the crystalline structures used to collect the data sets are identical. We note that these assumptions are only approximations, for the following reasons.

(i) The data sets are not necessarily collected at the same temperature. X-ray data are typically collected at cryo-temperatures (to avoid radiation damage), while neutron data are collected at room temperature. Data-collection temperature variations can lead to local and global differences in crystal structure, such as ionization states and hydrogen bonds (Fischer, 2021 ▸).

(ii) Neutron data collection takes longer (days or weeks) than X-ray data collection (minutes). It may be possible that the data-collection time impacts the resulting models, as the structures represent an average over space and time, *i.e.* an average of all unit cells of the crystal over the time span of the diffraction experiment.

(iii) The unit-cell parameters of crystals may differ slightly if different crystals are used for the experiments.

(iv) If the quality of the data sets differs (for example the resolution or completeness), the corresponding maps may show different amounts of detail. This means that if the model fits the higher quality data set, it will overfit the lower quality data. Conversely, a model that fits a poorer data set will underfit the better data.

(v) X-ray and neutron data sets convey different information about *X*—H^1^ bond lengths, because in the particular case of hydrogen the center of the electron distribution does not coincide with the position of the nucleus, but is systematically closer to the *X* atom along the covalent bond. As X-rays and neutrons interact with the electron cloud and the nuclei, respectively, the derived hydrogen positions will differ. As a result, *X*—H bonds are 10–20% shorter for H-atom positions derived with X-rays (Allen, 1986 ▸; Allen & Bruno, 2010 ▸).

(vi) If the diffraction data are collected from different crystals or at different data-collection temperatures, the solvent structure (water and ions) may be different.

Reports in the literature suggest that models from different data sets of the same protein differ, even when determined with the same diffraction method (X-ray). Two bovine trypsin–inhibitor complexes (Chambers & Stroud, 1979 ▸) and two models of transforming growth factor (Daopin *et al.*, 1994 ▸) exhibited root-mean-square differences (r.m.s.d.s) of 0.25 and 0.3 Å between C^α^ atoms, respectively. The largest differences were found in flexible regions in which the density of the residues was not clearly visible. A comparison of high-resolution structures from five crystals of bovine trypsin (obtained under analogous conditions) showed that while many details were similar, the side-chain orientations of residues located in flexible parts of the macromolecule varied significantly and about 25% of water-molecule positions were not conserved within 0.5 Å (Liebschner *et al.*, 2013 ▸).

We note that refinement against neutron data alone may mitigate most issues arising from refining one model against two data sets. Indeed, about one third of neutron models in the PDB were determined from neutron data alone. However, this approach may not be generally applicable. For example, Gruene *et al.* (2014 ▸) reported the refinement of models against high-resolution neutron data, but it is unclear how this approach performs at lower resolutions.

Therefore, we propose an alternative to the current joint XN procedure that avoids the assumption of strictly identical models for both diffraction experiments. We introduce a new joint XN refinement protocol that refines two models against the two data sets, *i.e.* an X-ray model against the X-ray data and a neutron model against the neutron data. The novel approach consists of using the model determined at higher resolution (typically the X-ray model) to generate reference-model restraints for the other model (typically the neutron model).

This work describes the rationale and application of our new joint XN procedure. We report how we prepared the models and the data used in the computations. We then show some properties of neutron models deposited in the PDB, which may be of interest to readers who are not experts in neutron macromolecular crystallography. The reason for developing the new joint XN procedure is that we assume that the crystalline structures used to collect the X-ray and neutron data are not necessarily identical. We test this assumption by separately refining four neutron models obtained by the conventional joint XN procedure against the X-ray and neutron data alone; we then compared the resulting two models (X-ray and neutron) to highlight the differences between them. To find out how to optimally refine H/D atoms in neutron structures, *i.e.* to determine whether it is advantageous to refine H/D atoms as individual or as ‘riding’ atoms, we performed test refinements of deposited neutron models against the neutron diffraction data alone. We then performed test refinements to exercise and validate our new joint XN procedure. To investigate whether the neutron models improved, we applied joint XN refinement to deposited neutron models obtained by conventional joint XN refinement. The neutron starting models were perturbed to increase the number of test structures and the reliability of the results. To analyze whether the X-ray models improved, we applied the new joint XN refinement protocol to deposited neutron models obtained by conventional joint XN refinement. Here, we did not apply perturbations to the X-ray starting models because we assumed that the deposited models were under-refined using the conventional approach.

## 2. Materials and methods

The computations were performed utilizing *Phenix* tools (Liebschner *et al.*, 2019 ▸) or custom scripts using the *Computational Crystallography Toolbox* (*cctbx*; Grosse-Kunstleve & Adams, 2003 ▸). To test the algorithms and procedures, we used neutron models and data deposited in the PDB for which we were able to reliably extract the atomic model and diffraction data. In some cases, we curated the models and/or data, as described in Section 3.1.

### 2.1. Obtaining models and data for the computations

For each neutron PDB entry, the following information was collected. The minimum and maximum resolution limits of the data were obtained from the PDB file header or, if absent, from the publication associated with an entry. For entries derived from neutron data alone, the reflection-array names in the data file followed the standard naming conventions in most cases. However, for joint XN data sets the data file should contain at least two data arrays: one for the neutron data and one for the X-ray data. In this case, there is unfortunately no naming convention that clarifies which array refers to which data set. To distinguish the arrays, we computed *R*_work_/*R*_free_ using X-ray and neutron scattering factors for both arrays, assuming that the wrong set of scattering factors leads to substantially higher values. Ligand restraints were obtained from the latest version of *GeoStd* (N. W. Moriarty & P. D. Adams, manuscript in preparation; https://github.com/phenix-project/geostd). In the case of uncommon ligand protonation, restraints were generated with *eLBOW* (Moriarty *et al.*, 2009 ▸). Also, if the recomputed *R* factors did not reproduce (within 5%) the values supplied by the authors of an entry, we checked for inconsistencies.

### 2.2. Collecting model and data properties

The entries derived from neutron diffraction experiments have inconsistent properties. Firstly, the models can originate from refinement against neutron data alone or from joint XN refinement. Secondly, the models can contain hydrogen, deuterium or both. If hydrogen and deuterium are present, a common scenario is that exchanged structures are modeled with hydrogen at nonlabile sites and a superposition of hydrogen and deuterium at labile sites. However, it is also possible to selectively protonate certain residues, such as the methyl groups of leucine and valine residues, as reported for example in Fisher *et al.* (2014 ▸). Finally, the diffraction data quality, such as the completeness and the resolution, can vary significantly. Therefore, it is useful to examine some properties of neutron entries before using them to test algorithms. To do this, we collected model properties, such as the hydrogenation state (how the experimentalists chose to model H-atom sites), and data properties, such as completeness and the resolution limit. We also estimated the number of unique neutron entries. As some studies involve the investigation of different mutants, ligand soaks *etc.* of the same molecule, the total number of neutron models does not reflect how many unique structures have been determined. For each neutron model, we obtained the sequences for all chains. If at least one sequence per model has more than 90% sequence identity to the chain of another model, the two structures are considered to be homologous. This way, we could determine groups (‘clusters’) of homologous models.

### 2.3. Refinement of models against X-ray and neutron data alone

To test our assumption that models refined separately against X-ray or neutron data are different, we separately refined four joint XN entries against the X-ray and neutron data: PDB entries 6l46, 7az3, 4ny6 and 3x2o. For refinement against neutron data, the deposited model was used. An exception is PDB entry 7az3, a model that contained either deuterated or hydrogenated residues, and which produced *R*_work_ and *R*_free_ values of 39.0% and 41.9%, respectively, for the neutron data if used as is. As the accompanying paper described the model as being perdeuterated, we replaced all non-exchangeable H atoms with D atoms and all labile sites with H/D, which yielded recomputed *R*_work_ and *R*_free_ values of 21.8% and 22.6%, respectively, which are much closer to the reported values (*R*_work_ and *R*_free_ of 18.4% and 22.1%, respectively). The reason for focusing on PDB entry 7az3 is because it has the highest neutron data completeness for a deuterated model.

For all four entries, refinement against X-ray data was performed with the deposited model in which all D and H/D sites were converted to single H sites. The refinement strategy was similar for both data sets: several macrocycles of reciprocal-space refinement with *phenix.refine* (Afonine *et al.*, 2012 ▸) that included refinement of individual coordinates, atomic displacement parameters (ADPs or *B* factors) and occupancies (as detailed in Afonine, 2015 ▸) and ordered solvent (water) update. As the resolution of the X-ray data was high enough, we refined the ADPs of non-H atoms anisotropically. After each round of refinement, the model was inspected with *Coot* (Emsley *et al.*, 2010 ▸) to validate and interactively improve the model. In the final rounds of refinement, we optimized the weights between the data and restraint targets (stereochemistry and ADP restraints).

### 2.4. Comparison of models refined separately against X-ray and neutron data

The *cctbx* library was used to compare the models that were refined separately against X-ray and neutron data. To compare the sites of water molecules, we used the water-cluster algorithm employed in the *Phenix Structure comparison* tool (Moriarty *et al.*, 2018 ▸). Water molecules were considered to be at the same site if they were within 0.5 Å. To compare the residues, we computed the rotameric states, calculated the coordinate r.m.s.d. after superposing the two models based on their main-chain atoms and identified whether a residue had alternative conformations. If a residue or a water molecule had a map–model correlation coefficient of less than 0.9 or 0.7, respectively, or if a water was modeled as an alternate conformation, it was disregarded. Also, if there was a significant negative difference map peak (less than −3 r.m.s.d.) along with weak average 2*mF*_obs_ − *DF*_model_ density (<1.7 r.m.s.d.) on atomic centers, the residue was ignored. In this way, we excluded uncertain parts of the model from the comparison. We also calculated histograms of isotropic ADPs to compare their distribution.

### 2.5. The new joint XN procedure in *phenix.refine*

In previous implementations of joint XN refinement, a single model was simultaneously refined against two data sets, thus inferring features that are common to both experiments. In the new joint XN approach, two models are refined against their respective data sets. The model refined against the X-ray data uses the shorter ‘X-ray’ *X*–H distances and the model refined against the neutron data uses the longer nuclear *X*–H distances. The refinements are not independent, however. The neutron model benefits from the details of the typically more accurate X-ray model via the use of reference-model restraints (Headd *et al.*, 2012 ▸). The ‘reference-model’ approach adds a restraint to each torsion angle (involving heavy atoms) in the working model; the target value of this restraint is set to the equivalent torsion in the reference model. The residuals for the reference torsion restraints use a ‘top-out’ function, which has the shape of a harmonic potential with an asymptotic threshold (‘top-out’). Unlike the procedure described in Headd *et al.* (2012 ▸), the joint XN approach does not perform automated correction of rotamer outliers in the working model. Similarly, the X-ray model retrieves the orientation of unambiguously resolved H atoms from the neutron model. Consequently, both models use their respective experimental data to the fullest extent, while also incorporating the unique features of each data set. Since the completeness and resolution of neutron data are almost always poorer than those of X-ray data (Figs. 2 ▸ and 3 ▸), it is expected that the neutron model benefits most from joint refinement.

### 2.6. Testing the new joint XN procedure

To exercise and validate the new joint XN procedure, we performed test refinements of perturbed neutron models against X-ray and neutron data. Using perturbed models offers three advantages. Firstly, the number of joint XN entries deposited in the PDB is very small (136 models), potentially obscuring trends in the interpretation of the refinement results. By using several perturbations of the same model, we can increase the number of test structures. Secondly, it can be assumed that deposited structures have been extensively validated and refined by the depositors, so it is unlikely that automated standard refinement procedures will improve the models substantially. This makes it more difficult to draw conclusions about the new procedure. However, as a perturbed model has been moved away from this optimized state, it is expected that it will improve if the refinement procedure is effective. Finally, since gradient-driven refinement is a local optimization procedure in a highly multi-dimensional space, it may occur that two similar refinement runs (for example using identical settings but slightly different initial models) converge to two slightly different refined models (Terwilliger *et al.*, 2007 ▸; Section 3.2.4 of Afonine *et al.*, 2018 ▸). The difference may manifest itself as changes in crystallographic *R* factors of as little as a fraction of a percent or as large as 1–2%. For this reason, the evaluation of refinement protocols based upon a single or a few refinements may not be reliable, and it is preferable to base conclusions on a large number of refinements.

Using *phenix.dynamics*, we created 20 perturbed versions for each joint XN model, applying r.m.s.d.s of 0.5 and 0.9 Å from the initial structure, yielding a total of 40 perturbations per model. These perturbations exceed the typical coordinate errors in refined models (see, for example, Rupp, 2009 ▸ and references therein) but are within the convergence radius of crystallographic refinement (Agarwal, 1978 ▸). The perturbed models were then refined (i) against the neutron data alone and (ii) using the new joint XN procedure. For the new joint XN refinement, the starting model for the neutron data was the perturbed model, while the starting model for the X-ray data was the deposited model (where deuterium was replaced with hydrogen). This scenario replicates the situation faced by an experimentalist, who in most cases has access to a good high-resolution X-ray structure and wants to determine the neutron model.

To investigate whether the new joint XN procedure systematically improves the X-ray models as well, we performed ten rounds of refinement. The starting model for the neutron data was the deposited model (curated if applicable); the starting model for the X-ray data was the deposited model where deuterium was replaced with hydrogen.

We then compared the water sites in the neutron and X-ray models from the new joint XN refinement procedure (with perturbation). We used the same water-cluster algorithm from the *Phenix Structure comparison* tool as was used to compare the four separately refined models (Section 2.4; waters were considered to be equivalent if within 0.5 Å). For this analysis, no map-filtering was applied.

We performed test refinements of all joint XN models that had both X-ray and neutron data available, that could be processed successfully and for which the recomputed *R* factors were within 5% of the published *R* factors.

### 2.7. Treating H (D) atoms as riding or as individual atoms

In addition to testing our new joint XN refinement procedure, we also investigated whether it is better to refine H (D) atoms as individual or as riding atoms. We recently reported the new riding hydrogen procedure employed in *cctbx* (Liebschner *et al.*, 2020 ▸). We apply a hybrid approach of this procedure for neutron refinement with *phenix.refine*: H atoms without a degree of freedom are treated as riding as described in Liebschner *et al.* (2020 ▸), while H atoms that possess a degree of freedom (such as O—H in serine or tyrosine) are allowed to refine freely in order to match the data. To test which approach is more advantageous, we refined 188 neutron models against their neutron data, once with the riding option and once with the individual option. We analyzed the results considering data completeness, data resolution and the H/D content of the sample.

## 3. Results and discussion

### 3.1. Obtaining models and data for the computations

As of April 2023, 212 neutron models had been deposited in the PDB. Among these, 136 models are from joint XN refinement and 76 models are derived from neutron data only, corresponding to a ratio of approximately 2:1. A total of 210 models could be successfully processed with *Phenix* tools. The two failures were due to uncommon ligand-protonation states. For example, a ligand in PDB entry 6bq8 was labeled as 6FW, but it actually had a different charge state than that implied by its chemical name. As a consequence, the restraints library file of 6FW had no restraints for a particular H atom in this ligand. After we contacted the PDB, the entry was updated (version 3.0) and the ligand was renamed WNU. As this change was very recent, the entry is not included in this study.

Several entries have no or incomplete diffraction data. Five models have no data at all (PDB entries 1gkt, 1io5, 1lzn, 1ntp and 6rsa). These entries predate the time when structure-factor deposition became mandatory for the PDB in 2008 (Young *et al.*, 2017 ▸). Seven joint XN entries have incomplete data arrays; the X-ray data are missing for PDB entries 5a93 and 3ins, while the neutron data are missing for PDB entries 5nfw, 5nfe, 6fjj, 6fji and 4cvj. Some of these entries are fairly recent (2019). This means that 5% of all neutron entries have no or incomplete data and cannot be validated or used for methods development.

We previously reported limitations to the annotation, deposition and validation of neutron models and data, which partly stem from a lack of community-wide accepted standards (Liebschner *et al.*, 2018 ▸). This created challenges for processing the remaining entries. For data files, for example, labels for joint XN entries could not always be allocated automatically, the *R*_free_ arrays were missing or incomplete (*i.e.* at least one reflection did not have an *R*_free_ flag) or the data annotation was incorrect (intensities versus structure factors). The most prominent issues with model files were related to H (or D) atoms. Some entries have the wrong atom type (H instead of D), some models had only one atom type at exchanged sites and some structures systematically missed certain H (or D) atoms. These problems could sometimes be identified by large differences between reported and recomputed *R* factors. We then modified the models according to information provided in the primary citation. For example, if the model contained only H atoms but the paper described the structure as being perdeuterated, we replaced all H atoms with D atoms. This time-consuming approach helped in some instances, but we still could not reproduce the X-ray or neutron *R*_work_ or *R*_free_, *i.e.* the recomputed values were 5% larger or smaller than those reported in the metadata, in 11 cases (PDB entries 2inq, 2mb5, 2vs2, 3kyy, 3kyx, 3qba, 4ar4, 5e5k, 5jpc, 5k1z and 5kwf).

### 3.2. Overview of some neutron model and data properties

Fig. 1 ▸ shows the annual cumulative number of neutron model depositions. The oldest neutron entry was deposited in the PDB in 1984. While the early numbers of deposited neutron models remained low (fewer than ten) until the early 2000s, depositions have been growing more rapidly since then. The increased rate of model depositions can be attributed to advanced neutron sources, new neutron macromolecular crystallography beamlines and improved methods and technologies, such as neutron image-plate detectors (Niimura *et al.*, 1994 ▸), as well as the use of powerful time-of-flight techniques (Langan *et al.*, 2004 ▸). We note that the majority of recent depositions come from joint XN refinements, although some models are derived from neutron data only.

Fig. 2 ▸(*a*) shows a histogram of the author-supplied high-resolution limit (*d*_min_) of the neutron data. The best resolution is 1.05 Å for PDB entry 4ar3 (Cuypers *et al.*, 2013 ▸) and the lowest resolution is 2.8 Å for PDB entry 8e1w (Tandrup *et al.*, 2023 ▸). For all neutron entries the average high-resolution limit is 1.99 Å, while for joint XN and only neutron entries the average *d*_min_ amounts to 2.04 and 1.91 Å, respectively. In addition, we observe that the distribution of *d*_min_ for only neutron models is slightly shifted towards higher resolutions, suggesting a tendency to use the joint XN approach for lower neutron data resolutions.

Fig. 2 ▸(*b*) shows the relationship between the high-resolution limits of neutron and X-ray data for joint XN entries. In the majority of cases the X-ray data resolution is better than that of the neutron data. This is expected for several reasons (also discussed in Section 1). Firstly, X-ray diffraction experiments are usually carried out at cryo-temperatures, which decreases the intensity decay of the high-resolution Bragg reflections. Secondly, the samples used for neutron diffraction underwent perdeuteration or soaking, which often results in smaller, less well diffracting crystals.

Fig. 3 ▸(*a*) shows a histogram of the completeness of the neutron data. The worst completeness is 45.5% for PDB entry 5kwf (Golden *et al.*, 2017 ▸) and the best completeness is 99.7% for entry 5ai2 (Cuypers *et al.*, 2016 ▸). For all neutron entries, the average completeness is 81.8%; for joint XN entries and only neutron entries, the average completeness values are 80.9% and 83.3%, respectively. For the high-resolution limit, we observed a slight shift towards higher completeness in the distribution of only neutron models. Fig. 3 ▸(*b*) shows the distribution between the completeness of neutron and X-ray data for joint XN entries. In most cases, the completeness of the X-ray data surpasses that of the neutron data.

**Figure 4:**
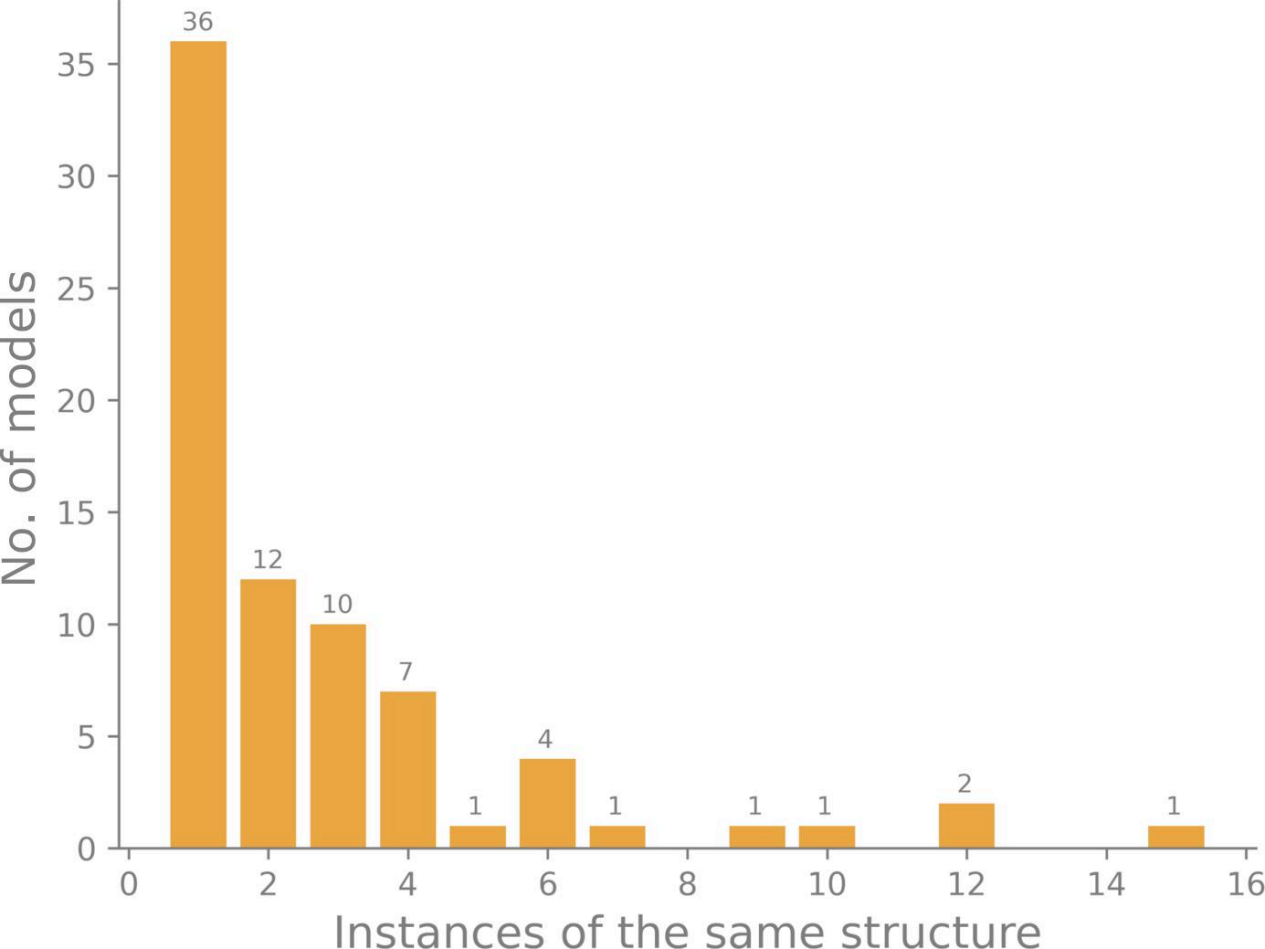
Histogram of the number of unique models determined by neutron diffraction.

Fig. 4 ▸ illustrates how many similar structures have been determined using neutron diffraction. Based on the sequence, we assigned each neutron model to a cluster of similar structures. The *x* axis represents the number of models in a cluster; if the number is one, there is only one instance of the structure in the PDB. Larger numbers mean that several versions of a structure have been determined. The height of the bar denotes how many clusters exist. For example, there are 36 structures that have only one instance of a model in the PDB, 12 structures that have two instances *etc.* Some structures have been determined many times: there are 15 models of trypsin, 12 models of rubredoxin and 12 models of human carbonic anhydrase II. This means that the 212 neutron models in the PDB contain many homologous structures. While this finding does not immediately affect the computations reported in this work, it is worthwhile noting this property.

### 3.3. Comparison of four models refined separately against X-ray and neutron data

To test our hypothesis that one single model cannot simultaneously satisfy the X-ray and neutron diffraction data in the best manner, we chose four joint XN models (PDB entries 6l46, 7az3, 3x2o and 4ny6) for which we performed refinements separately. To be able to compare structural details, the neutron and X-ray data needed to be of good quality, so this was our main criteria in choosing these entries. Statistics of the deposited and re-refined models for these four examples are summarized in Table 1 ▸. The resolution of the neutron data was 1.5, 1.7, 1.5 and 1.85 Å for PDB entries 6l46, 7az3, 3x2o and 4ny6, respectively. We note that the paper describing PDB entry 6l46 (Fukuda *et al.*, 2020 ▸) reports a resolution limit of 1.3 Å for the X-ray data for their analysis, because it ‘yielded better *R* factors and less noise’. As the deposited data set included reflections (with 99.9% completeness) up to 1.0 Å resolution, we used the full resolution range. Notably, we did not observe any abnormalities in the maps or in refinement. Another criterion for choosing these particular entries was the comparably high completeness of the neutron data, ranging from 88.1% (PDB entry 7az3) to 99.6% (PDB entry 6l46), which is markedly better than the average of 81.8% (Section 3.2). This way, the resulting models should be quite accurate and well suited for a detailed comparison.

If the model was refined against the X-ray data alone, *R*_work_ and *R*_free_ improved in all cases. For PDB entry 4ny6, the improvement amounts to more than 5%. For the model refined against neutron data alone, the *R* factors are slightly better than or similar to the values referenced in the PDB.

**Figure 5:**
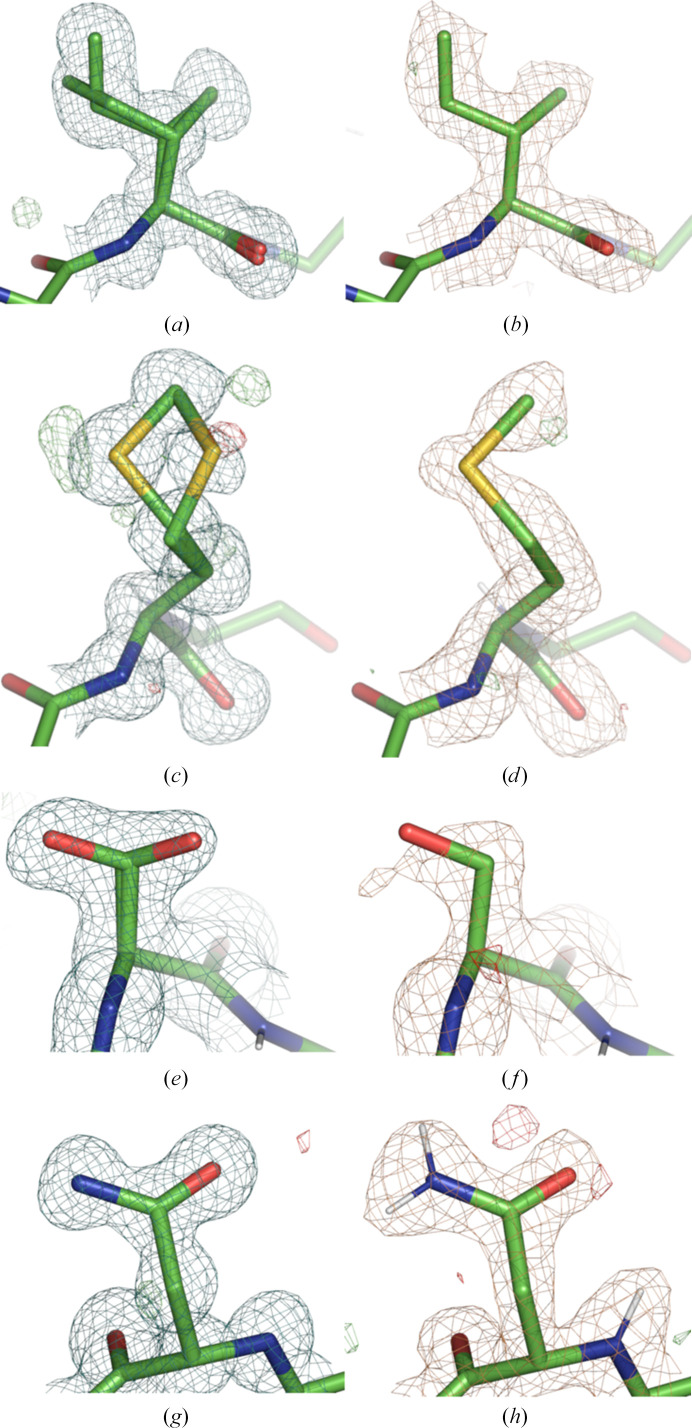
Examples of different numbers of alternative conformations and how an asparagine residue appears in electron-density and nuclear scattering length density maps. In all figures gold mesh represents neutron scattering length density and teal mesh represents electron density. Green/red represents *mF*_obs_ − *DF*_model_ density contoured at 3.0/−3.0 r.m.s.d., carved at 3 Å. (*a*, *b*) Ile52 in PDB entry 6l46. Note that there are no notable peaks in the difference density maps around this residue. (*c*, *d*) Met266 in PDB entry 6l46. (*e*, *f*) Ser160 in PDB entry 3x2o. (*g*, *h*) Asn38 in PDB entry 3x2o. The 2*mF*_obs_ − *DF*_model_ density is contoured at 0.8 r.m.s.d. in (*a*), (*b*), (*c*) and (*d*), 1 r.m.s.d. in (*e*) and (*f*) and 2 r.m.s.d. in (*g*) and (*h*). Side-chain H/D atoms are not shown for clarity, except for the Asn group in (*h*).

Comparing each residue one by one revealed that each X-ray model had more alternative conformations than the corresponding neutron model. The number of additional alternative conformations ranged from 17 (PDB entry 4ny6) to 34 (PDB entry 6l46). Examples of alternative conformations are shown in Fig. 5 ▸. The first example shows Ile52 in PDB entry 6l46. The electron density clearly (Fig. 5 ▸ *a*) supports the two alternative conformations, while the nuclear scattering length density only shows one conformation (Fig. 5 ▸ *b*). Another example is Met266 in PDB entry 6l46. Methionine contains an S atom, which is a strong scatterer of X-rays [*f*(0) = 16 e^−^] but a weak scatterer of neutrons (*b* = 2.8 fm). The electron density supports the two modeled conformations (Fig. 5 ▸ *c*). The difference density peaks around the S and C atoms of Met266 may be indicative of a third conformation or of radiation damage. In contrast, there are no peaks in the nuclear scattering length density map (Fig. 5 ▸ *d*) that would justify modeling the second conformation present in the X-ray model. A third example is Ser160 in PDB entry 3x2o, the double conformation of which is clear in the X-ray model (Fig. 5 ▸ *e*), while only one conformation appears in the neutron model (Fig. 5 ▸ *f*). Asn38 in PDB entry 3x2o is an example for which the nuclear scattering length density map may be more informative. As nitrogen and oxygen have a similar number of electrons at zero scattering angle, they are often difficult to distinguish by the density map alone. The orientation of Asn and Gln side chains is thus often ambiguous, so that clashes and hydrogen-bond interactions with neighboring entities have to be taken into account. However, if the H atoms of the Asn head group are replaced by D atoms, the NH2 (ND2) group is a much stronger scatterer than the O atom, so that the side-chain orientation can be determined from the nuclear scattering length density map. It is thus conceivable that the orientations of Asn and Gln residues could be determined from the neutron data instead of the X-ray data. While this is not currently implemented in our new joint XN refinement procedure, it represents a future avenue of improvement.

A remarkable structural difference involved the number of water molecules. All X-ray models had more water molecules than the neutron models. The number of water molecules in the deposited structures was generally between the two values. For example, in the case of PDB entry 7az3, the PDB model had 203 waters, the X-ray model contained 230 waters and the neutron model had 156 waters. We also note that the coordinates of a significant part of the water molecules were not conserved. We determined which water molecules were within 0.5 Å in both models, and those that did not have a corresponding water were considered to be ‘lone’ waters. The percentage of lone waters was generally higher in the X-ray models, which is consistent with the observation that these models generally had more water molecules. This percentage varied between 27% (PDB entry 6l46) and 81% (PDB entry 4ny6). However, even the neutron models had many lone waters, *i.e.* between 17% (PDB entry 6l46) and 58% (PDB entry 4ny6). We note some correlation of the percentage of lone waters with the data-collection temperature. PDB entry 6l46 has the lowest percentage of lone waters and both the neutron and X-ray data were collected at 100 K. PDB entries 7az3 and 3x2o have 43% and 49% lone waters in the X-ray model, respectively, and the data were collected at room temperature in both cases.

For PDB entry 4ny6, the percentage of lone waters is the highest (81% for X-ray and 58% for neutron). While both the neutron and X-ray data sets were obtained at room temperature, they were collected from different crystals. The neutron and X-ray data stem from a selectively deuterated crystal (valine residues and methyl groups of leucine) and a perdeuterated crystal, respectively. The authors state

The assumption here is that the X-ray data from the selectively protonated deuterated crystals should be the same as those from the perdeuterated crystal, as H and D are equivalent using X-rays

(Fisher *et al.*, 2014 ▸). While H and D are equivalent with either diffraction method, our analysis shows that the water structure varies between the two data sets that were collected from different crystals.

**Figure 6:**
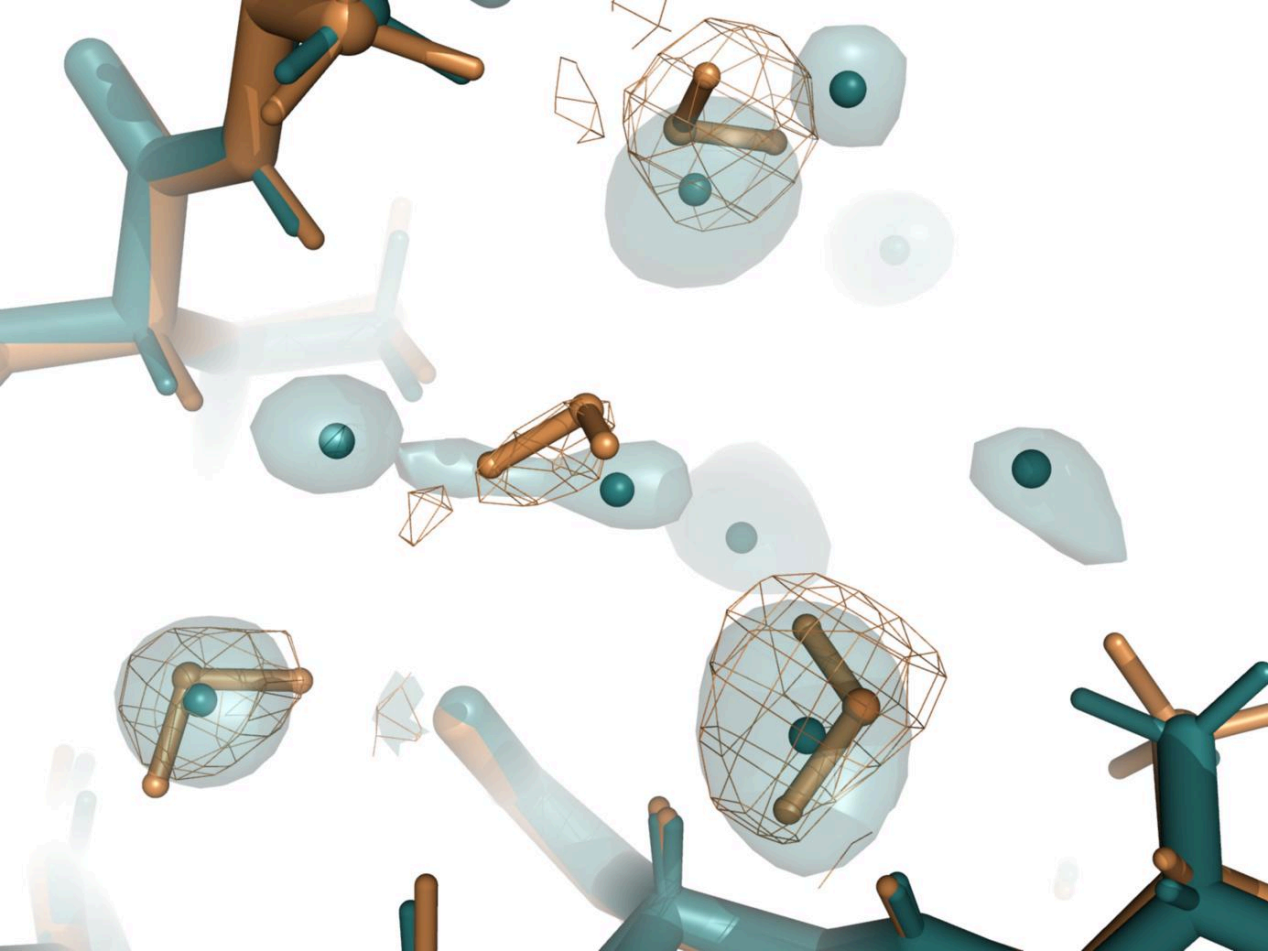
Example of differing positions of water molecules. Water molecules in X-ray (teal) and neutron (gold) models of entry 4ny6 are shown. The 2*mF*_obs_ − *DF*_model_ density is contoured at 0.8 r.m.s.d.; gold mesh, neutron scattering length density; teal surface, electron density.

As an example of the differing water molecules between X-ray and neutron models, Fig. 6 ▸ shows the region between two symmetry-related molecules of PDB entry 4ny6. The four DOD molecules have corresponding HOH O atoms of the X-ray model in their vicinity. However, even if the O atoms are within 0.5 Å, their coordinates differ visibly. Further, there are several water molecules in the X-ray model that do not have an equivalent in the neutron model. Notably, there are no 2*mF*_obs_ − *DF*_model_ peaks indicating the presence of the DOD molecules near the position of the X-ray waters.

**Figure 7:**
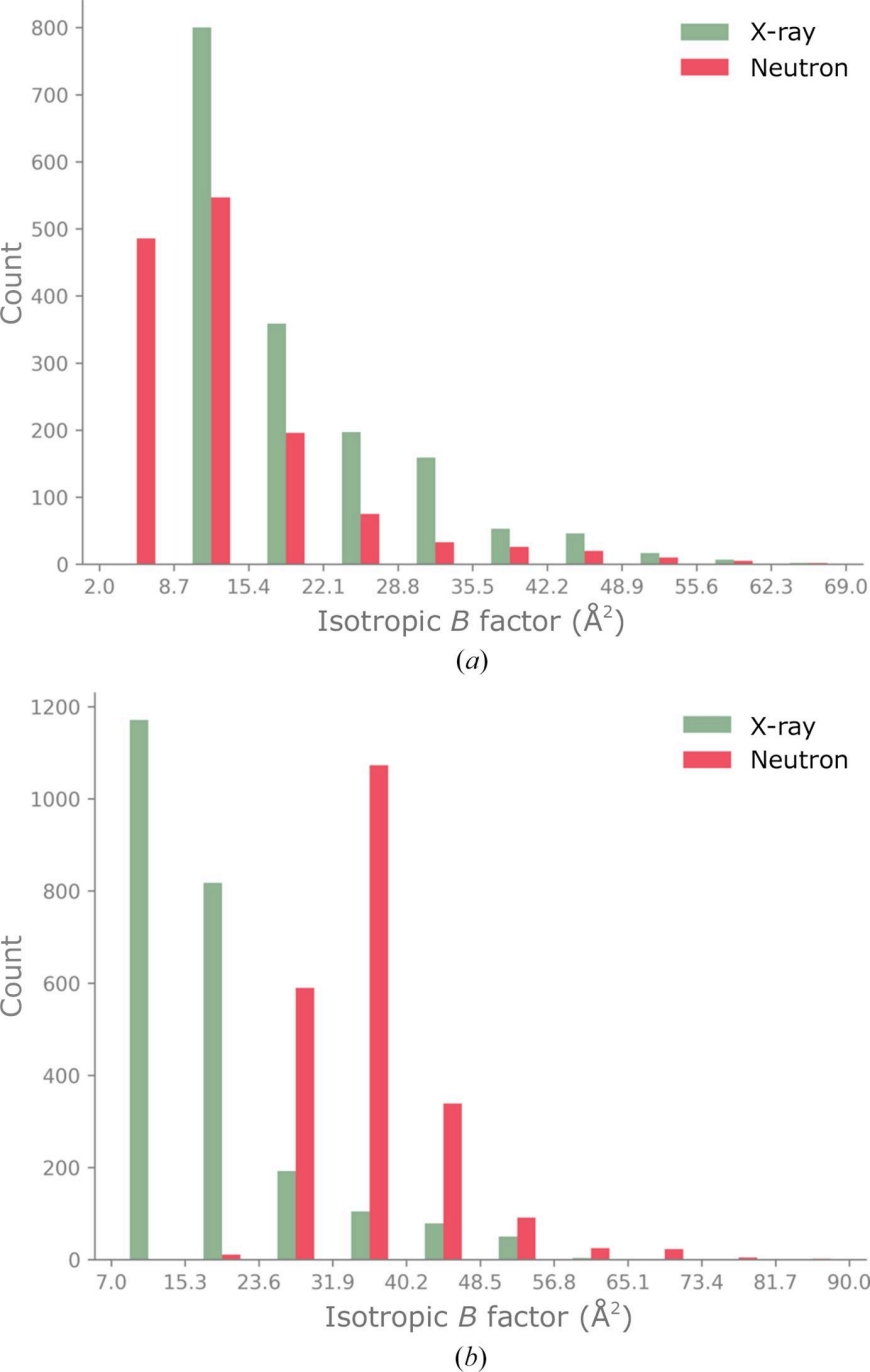
Histograms of isotropic ADPs in (*a*) PDB entry 3x2o and (*b*) PDB entry 7az3.

We also analyzed the isotropic ADPs (*B*_iso_) of the non-H (non-D) atoms in the models. The minimum, maximum and mean values for protein residues and water molecules are listed in Table 1 ▸. We note that the average *B*_iso_ of protein residues is lower in most neutron models, with the exception being PDB entry 7az3. This entry is the only perdeuterated model among the four examples, but it is unclear whether this is the reason for the behavior of the ADPs. We also made ADP histograms to investigate their distribution. The histograms are comparable for the X-ray and neutron models of PDB entries 6l46 and 4ny6. The histograms for PDB entries 3x2o and 7az3 are shown in Fig. 7 ▸. In both cases, the distributions are markedly different. For PDB entry 3x2o the histogram of ADPs is shifted to lower values, while for PDB entry 7az3 it is shifted to larger values. It is obvious that one single model cannot simultaneously satisfy both respective ADP distributions.

Finally, we computed crystallographic *R* factors from the X-ray data and the neutron models in which all D atoms were replaced with H atoms (water H/D atoms were removed). These *R* factors are significantly different from those obtained from refining a model against X-ray data. For example, in the case of PDB entry 4ny6, the recomputed *R*_work_ and *R*_free_ are 28.7% and 30.8%, respectively, and are much worse than those from the re-refined X-ray model (11.1% and 13.1%, respectively). This means that a model that fits the neutron data best will not do the same with the X-ray data. We note that the reverse computation, *i.e.* calculating *R* factors for the X-ray model against the neutron model, will not be very informative unless the protonation states of the neutron model are replicated. Transferring the protonation states may be possible for residues and waters present in both models, but will prove challenging for entities that can only be found in the X-ray model. If the protonation is incorrect, the difference in *R* factor will not reflect model differences, but issues with H/D atoms.

Overall, this comparison showed that significant differences exist between models refined separately against neutron and X-ray data, with the largest difference being in the water structure and isotropic ADPs.

### 3.4. Refining H (D) as riding or as individual atoms

**Figure 8:**
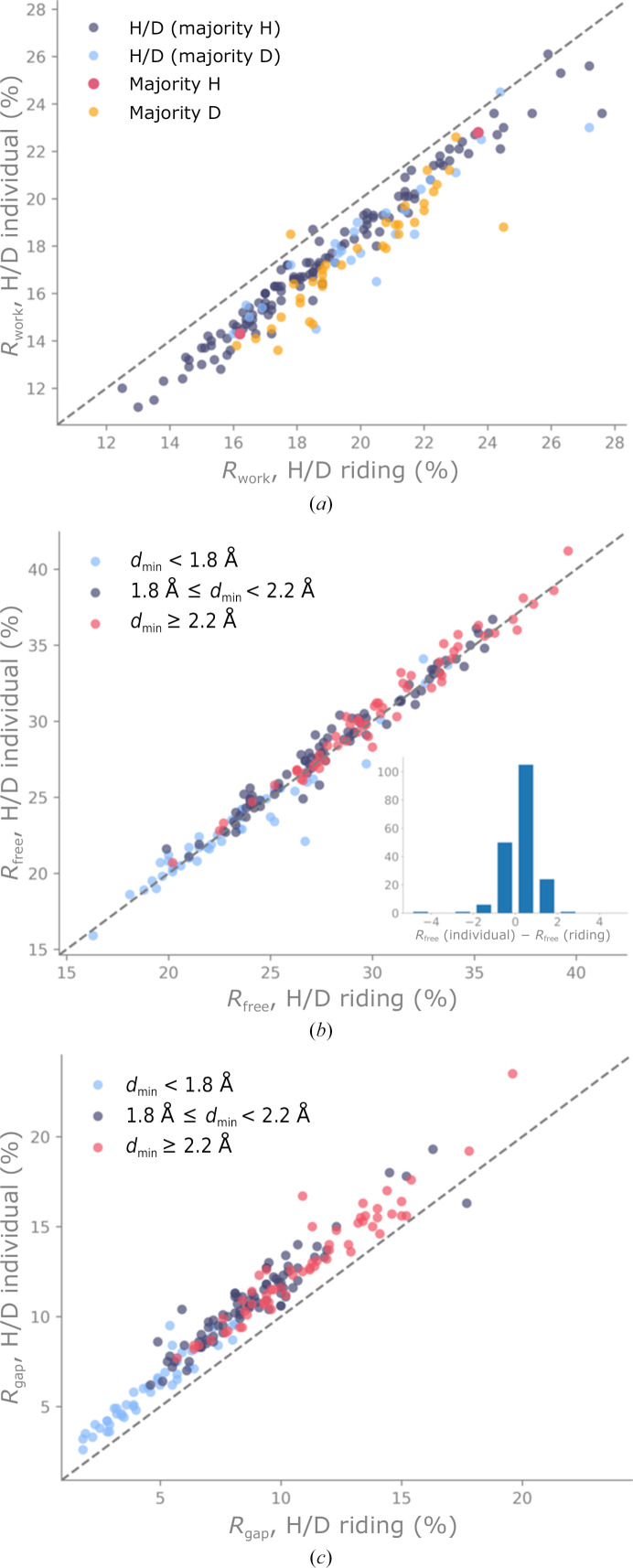
(*a*) *R*_work_, (*b*) *R*_free_ and (*c*) *R*_gap_ for refinements against neutron data only using either the riding or the individual option for H/D atoms. The points are colored according to properties of the data or model, according to the property that showed the most correlation. The gray dashed line represents the bisector.

We investigated whether it is more advantageous to refine H/D atoms against neutron diffraction data as riding or as individual atoms. Refinements were successfully completed for 188 neutron models. We analyzed the results considering the data completeness, the high-resolution limit of the neutron data and the H/D content of the sample. Fig. 8 ▸ shows *R*_work_, *R*_free_ and *R*_gap_ (*R*_free_ − *R*_work_) for refinements using either the riding or individual option for H/D atoms. If the distribution was dependent on one of the three properties considered (completeness, resolution or H/D content), we colored the scatter points accordingly.

Fig. 8 ▸(*a*) shows the crystallographic *R* factor *R*_work_. Almost all of the points of the scatter plot are below the bisector, meaning that refinement leads to a lower *R*_work_ if H/D atoms are refined individually. This behavior is unsurprising because, generally, increasing the number of model parameters typically decreases *R*_work_. The points in Fig. 8 ▸(*a*) are colored according to the hydrogenation state. We observe that points representing models with a majority of D atoms (perdeuterated models) are often furthest from the bisector, *i.e.* they have a lower *R*_work_. We did not observe a dependence on completeness or on the resolution limit.

Fig. 8 ▸(*b*) shows the crystallographic *R* factor *R*_free_. Most points of the scatter plot are above the bisector, meaning that refinement leads to slightly higher *R*_free_ if H/D atoms are refined individually. As the points are generally close to the bisector, we also plotted a histogram of *R*_free_(individual) − *R*_free_(riding) (see the inset in Fig. 8 ▸ *b*). The distribution is shifted to the right, and thus shows that the strategy of refining H/D atoms individually results in an increased *R*_free_ in many cases. The points in Fig. 8 ▸(*b*) are colored according to the high-resolution limit. We observe that neutron data with a lower resolution limit *d*_min_ result in a higher *R*_free_. We did not observe a dependence on completeness or on the hydrogenation state.

Fig. 8 ▸(*c*) shows the difference between the *R* factors, *R*_gap_. All points except one are above the bisector, which means that *R*_gap_ is lower if the H/D atoms of the model are refined with the riding model. This is in line with the results from Figs. 8 ▸(*a*) and 8 ▸(*b*), where *R*_free_ was lower and *R*_work_ was higher when H/D atoms were refined as riding. The points in Fig. 8 ▸(*c*) are colored according to the high-resolution limit. The distribution of the points depends on the resolution of the data: data with better resolution (better than 1.8 Å) yield a lower *R*_gap_ than data with poorer resolution (worse than 2.2 Å). We also observed a tendency of refinements to result in a lower *R*_gap_ (not shown) if data completeness is high, but the dependence was less apparent than for the high-resolution limit. We did not observe a dependence on the hydrogenation state.

Together, the results shown in Fig. 8 ▸ suggest that refining individual parameters of H/D atoms leads to overfitting unless the high-resolution limit of the data is high. As a general recommendation, we suggest that if the resolution of the neutron data is better than 2 Å, both strategies should be tried to find out whether H/D atoms in the model should be refined as riding or individually. At low resolution and low completeness, it can be assumed that the riding model is adequate.

**Figure 9:**
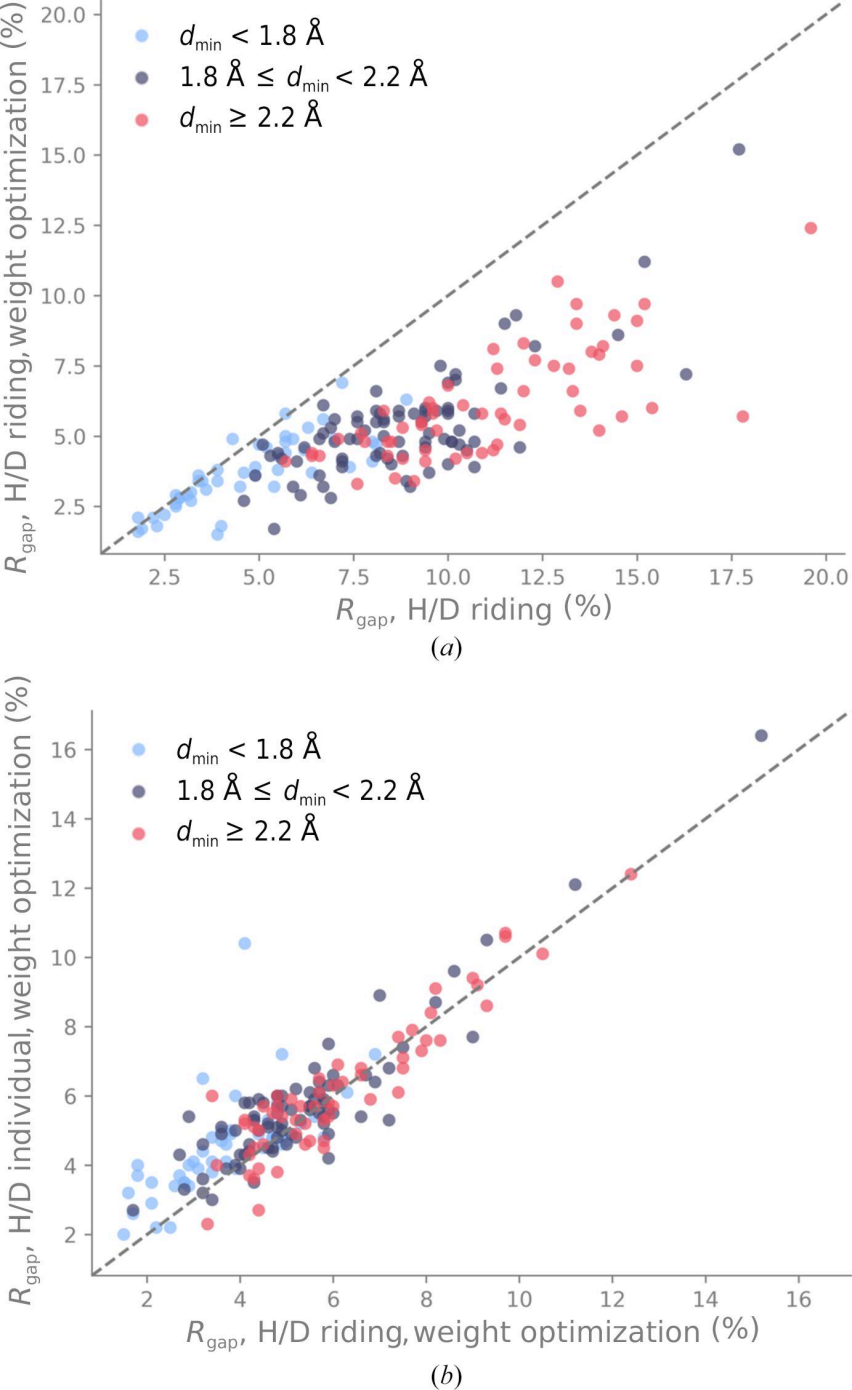
(*a*) Comparison of *R*_gap_ with and without weight optimization for the riding model. (*b*) *R*_gap_ for refinements using either the riding or the individual option and with weight optimization for both.

We performed additional refinements, this time also optimizing the weights between the data target and the stereochemistry and ADP restraints, respectively (Afonine *et al.*, 2011 ▸). This weight-optimization procedure tries to find the weights that result in the lowest *R*_free_ factors by performing a grid search over an array of weight candidates. Fig. 9 ▸(*a*) shows a comparison of *R*_gap_ with and without weight optimization for the riding model. We observe two things. Firstly, the distribution is shifted towards lower values of *R*_gap_ compared with Fig. 8 ▸(*c*), meaning that weight optimization can decrease the difference between *R*_work_ and *R*_free_. Secondly, the behavior of the distribution depends on the resolution of the neutron data. For high-resolution data, *R*_gap_ is similar with or without using the weight-optimization procedure. At lower resolutions, the weight optimization clearly leads to a lower *R*_gap_. Therefore, it seems advantageous to optimize the weights if the neutron data resolution is low. Fig. 9 ▸(*b*) shows *R*_gap_ computed for refinements with weight optimization using either the riding or the individual option. Again, the distribution is shifted towards lower values of *R*_gap_ compared with Fig. 8 ▸(*c*). Furthermore, the distribution is more centered around the bisector for data with poorer resolution limits (worse than 1.8 Å) than for high-resolution data. If the weights are optimized, there is less difference between the *R*_gap_ values for riding versus individual H/D refinements, except for neutron data with high resolution. Altogether, the results in Fig. 9 ▸ suggest that weight optimization mitigates the effects of overfitting at lower neutron data resolution limits.

### 3.5. Testing the new joint XN procedure: neutron models

**Figure 10:**
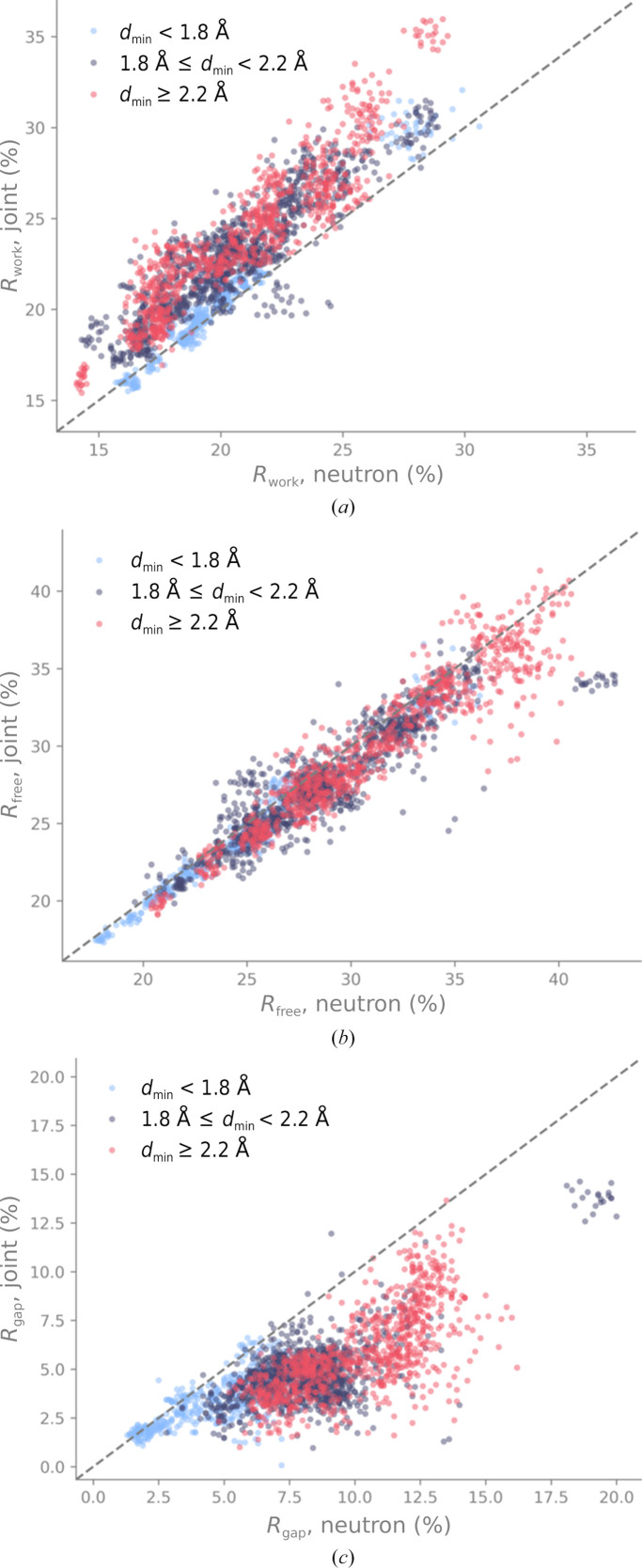
(*a*) Neutron *R*_work_, (*b*) *R*_free_ and (*c*) *R*_gap_ for refinements of models perturbed at 0.5 Å against neutron data only or using the new joint XN approach. The points are colored according to the high-resolution limit of the neutron data. The gray dashed line represents the bisector. The separate cluster of points (also present in Fig. 11 ▸) corresponds to perturbations of one particular model for which manual refinement and curation may be required to produce better refinement outcomes.

Of the 136 joint XN entries, 120 had both X-ray and neutron data available, could be processed successfully and had recomputed *R* factors within 5% of the published *R* factors. For each of these 120 models, we created 40 perturbations (20 at a threshold of 0.5 Å and 20 at a threshold of 0.9 Å). Each perturbed model was refined against neutron data alone or used as the neutron starting model for a new joint XN procedure. This gave 2400 computations per threshold (2400 = 20 × 120). 2393 and 2390 refinements ran to completion at thresholds of 0.5 and 0.9 Å, respectively. Fig. 10 ▸ shows *R*_work_, *R*_free_ and *R*_gap_ from refinements against neutron data only against those from new joint XN refinements, for models perturbed with a threshold of 0.5 Å. The points are colored according to the high-resolution limit of the neutron data.

Fig. 10 ▸(*a*) shows the crystallographic *R* factor *R*_work_. Most points of the scatter plot are above the bisector, meaning that *R*_work_ tends to be lower if the starting model is refined against the neutron data only. We observe that some data points in the lower *R*_work_ region, mostly those corresponding to models determined at better than 1.8 Å resolution (blue circles), are located close to or on the bisector, *i.e.* *R*_work_ is similar for both refinement approaches.

Fig. 10 ▸(*b*) shows the crystallographic *R* factor *R*_free_. Many points of the scatter plot are below the bisector, *i.e.* *R*_free_ tends to be lower for the new joint XN refinements. We again observe a dependence of the distribution on the high-resolution limit. *R* factors from high-resolution data (better than 1.8 Å, blue circles) occupy the regions of lower *R*_free_ and are arranged close to the bisector, while those from lower resolution data (worse than 2.2 Å, red circles) are in the regions of higher *R*_free_ and display more scatter around the bisector.

Fig. 10 ▸(*c*) shows the difference between the *R* factors, *R*_gap_. The majority of points are below the bisector, indicating that new joint XN refinement minimizes *R*_gap_. As large differences between *R*_work_ and *R*_free_ are typically associated with overfitting, it can be concluded that the new joint XN approach reduces the overfitting of models compared with refinement against neutron data alone. The effect is less pronounced for high-resolution data (blue circles), *i.e.* the new joint refinement or refinement against neutron data leads to similar *R*_gap_.

**Figure 11:**
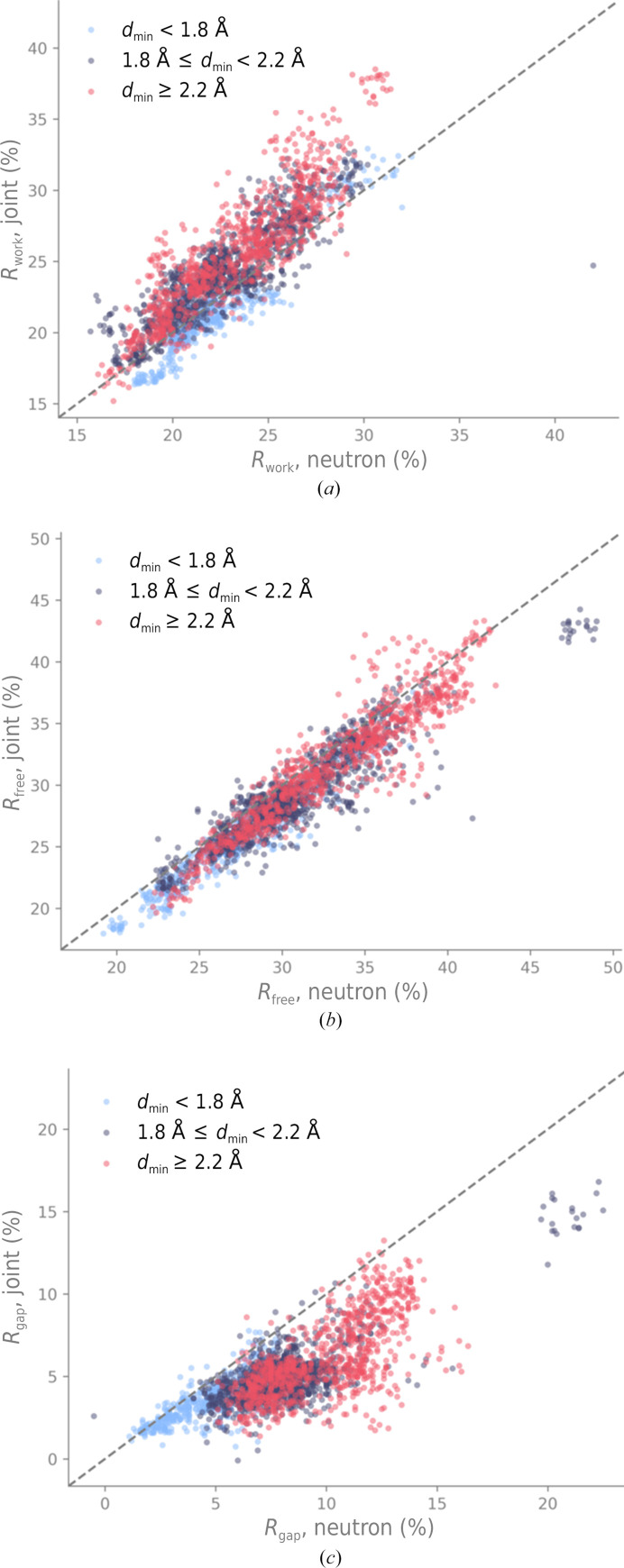
(*a*) Neutron *R*_work_, (*b*) *R*_free_ and (*c*) *R*_gap_ for refinements of models perturbed at 0.9 Å against neutron data only or using the new joint XN approach. The points are colored according to the high-resolution limit of the neutron data. The gray dashed line represents the bisector.

Fig. 11 ▸ shows *R*_work_, *R*_free_ and *R*_gap_ after refining models perturbed with a threshold of 0.9 Å. The shape of the distribution and the dependence on the high-resolution limit of the neutron data is very similar to that of the *R* factors obtained for the 0.5 Å perturbations. The conclusions to be drawn are therefore the same: the joint XN approach reduces overfitting, especially when the neutron diffraction data have medium-to-low resolution.

**Figure 12:**
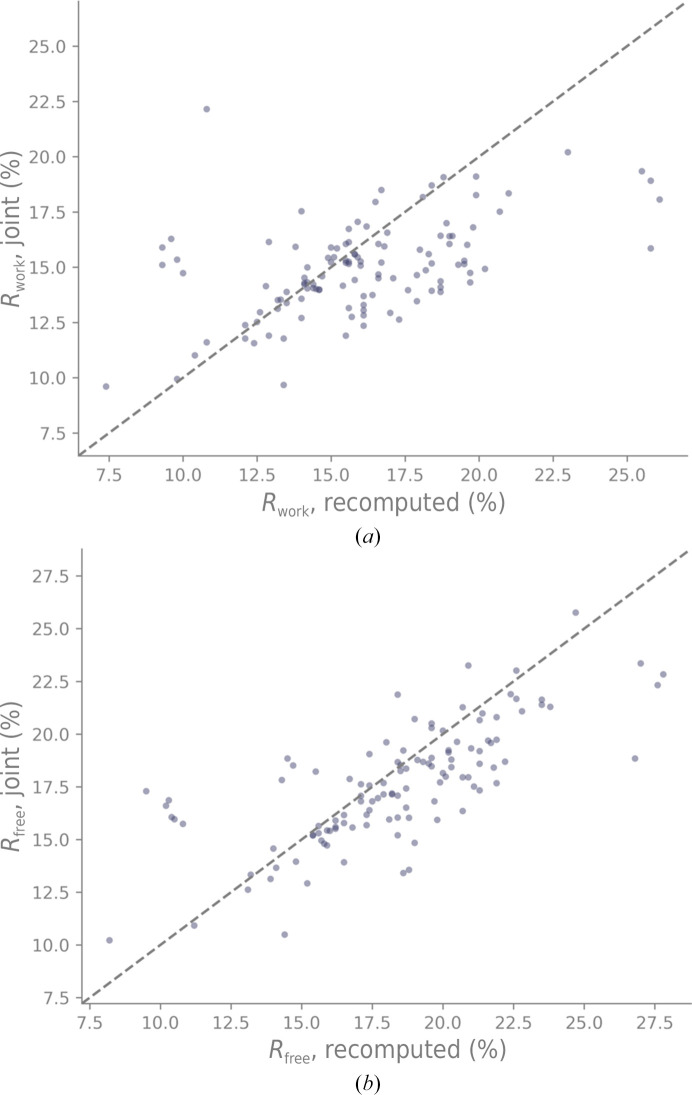
X-ray (*a*) *R*_work_ and (*b*) *R*_free_ recomputed from deposited neutron models against refinements using the new joint XN approach (the starting model was not perturbed). The gray dashed line represents the bisector.

We also investigated whether the new joint XN procedure systematically improves the X-ray models. These computations were carried out with the deposited models (curated if applicable) where D was replaced with H for the X-ray starting model. Our assumption was that the deposited models most likely under-refined the X-ray data, so the new joint XN approach should naturally lead to improvements. Figs. 12 ▸(*a*) and 12 ▸(*b*) show the crystallographic *R* factors *R*_work_ and *R*_free_ recomputed from the deposited models and after the new joint XN refinement, respectively. Most points of the scatter plot are below the bisector, meaning that the *R* factors are lower if the model is refined against the X-ray data only.

**Figure 13:**
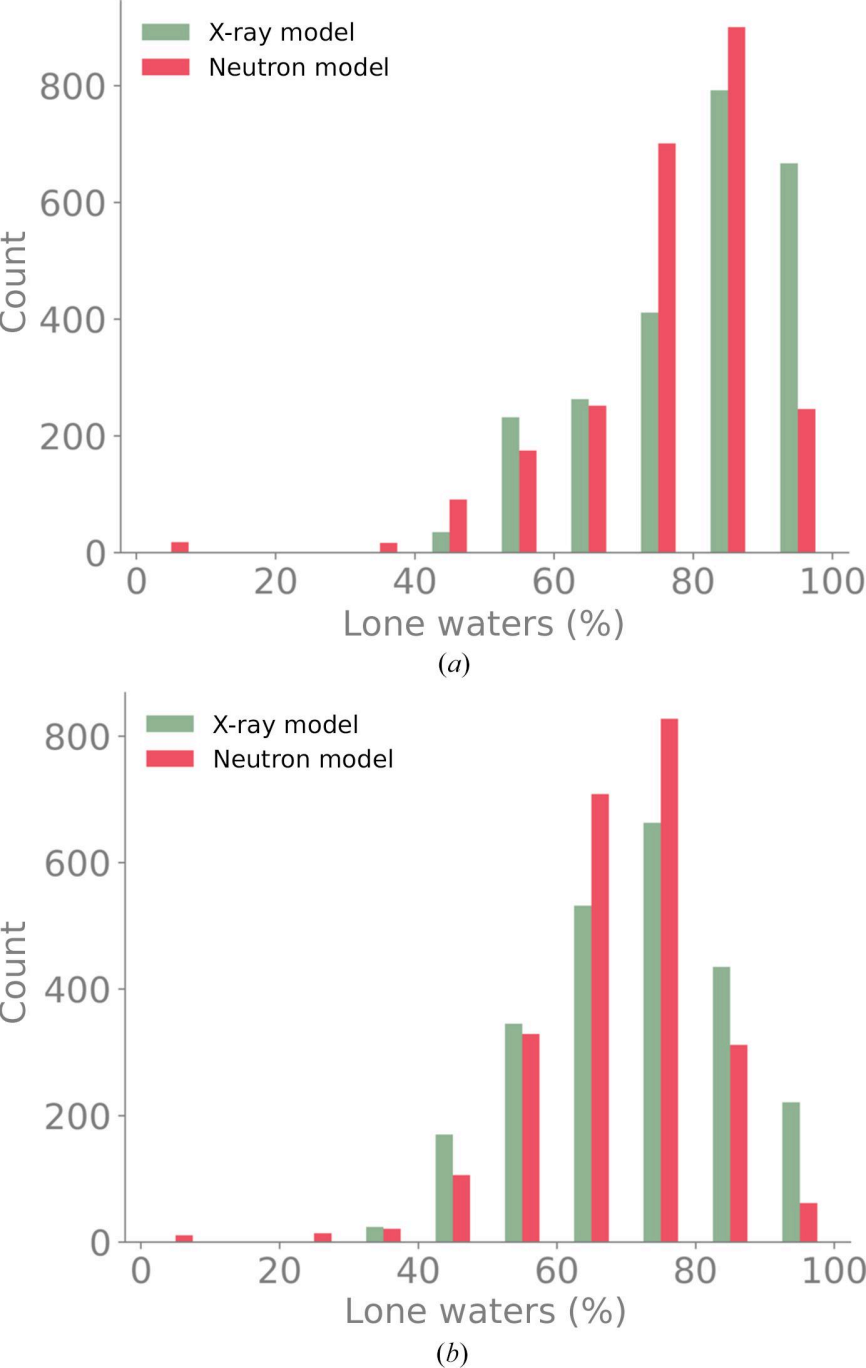
Histogram of the percentage of lone waters (water molecules without an equivalent) for the neutron and X-ray models after the new joint XN refinement. (*a*) Refinements from 0.9 Å perturbations. (*b*) Refinements from 0.5 Å perturbations. Some models had no waters placed by the ordered solvent procedure in the new joint XN refinement procedure. These correspond to the bar in the 0–10% bin.

As analysis of the individually refined models showed that the most significant difference concerned the sites of water molecules (Section 3.3), we compared the coordinates of waters in the neutron and X-ray models from the new joint XN refinement. Fig. 13 ▸ shows a histogram of the percentage of lone waters (from the refinement of perturbed neutron models), *i.e.* water molecules in one model that do not have an equivalent in the other model, for the two perturbation levels (0.9 Å in Fig. 13 ▸ *a* and 0.5 Å in Fig. 13 ▸ *b*). At the 0.9 Å perturbation level the histogram is centered on large percentage values, *i.e.* X-ray models have 70–100% lone waters, while neutron models have 70–90% lone waters. At smaller perturbations the percentage is centered between 60% and 80% for either model. This means that a significant number of water molecules do not occupy the same sites in the two models. Our new joint XN refinement approach is therefore justified as it allows the two models to have different water structures.

## 4. Conclusions

While neutron entries represent only a small fraction of the models deposited in the PDB, they provide unique and important information for understanding biological function because neutron scattering length density maps can visualize the positions of H atoms. As H atoms can be refined against the neutron diffraction data along with the non-H atoms, the number of parameters to be refined increases substantially. This may lead to overfitting, which is exacerbated by the typical deficiencies of neutron diffraction data, such as lower completeness and resolution. We investigated two strategies that can compensate for these issues. Firstly, we analyzed the adequacy of refining the H (D) atoms as riding or as individual atoms. We found that unless the data resolution is very high, the riding model reduces overfitting. Weight optimization may alleviate this overfitting somewhat. Secondly, we developed a new procedure for joint XN refinement that optimizes two models against their respective neutron and X-ray data sets, with one model supplying reference restraints for the other. To illustrate why it is advantageous to use two models, we separately refined four joint XN entries from the PDB against their X-ray and neutron data and compared the resulting models. We found that significant differences exist between the X-ray and neutron models, supporting our premise that joint XN refinement should be carried out with two separate models. We then performed tests of the new joint XN procedure against a large number of perturbed joint XN models. The analyses showed that the new joint XN refinement reduces overfitting at low-to-medium neutron data resolution.

## Figures and Tables

**Table 1:** Statistics of the deposited and re-refined models for four joint XN examples from the PDB

PDB code		6l46	7az3	3x2o	4ny6
Data-collection temperature (K)	X-ray	100	293	298	298
	Neutron	100	293	298	298
Resolution (Å)	X-ray	1.00 (1.3)†	1.15	1.00	1.05
	Neutron	1.50	1.70	1.50	1.85
Completeness‡ (%)	X-ray	99.9	99.5	99.3	99.8
	Neutron	99.6	88.1	93.5	90.0
No. of protein residues		298	248	180	65
No. of waters		458	203	152	42
*R*_work_/*R*_free_					
Deposited (%)	X-ray	9.7/11.2	14.0/15.3	13.5/15.1	17.0/18.8
	Neutron	14.2/15.9	18.4/22.1	22.8/25.1	17.6/22.5
Re-refined (%)	X-ray	8.8/9.7	9.8/11.4	8.9/9.9	11.1/13.1
	Neutron	13.0/15.7	18.8/22.6	21.4/25.0	17.3/23.3
Neutron model against X-ray data (%)		17.0/16.9	24.5/24.8	22.0/22.9	28.7/30.8
R.m.s.d. (Å)		0.048	0.089	0.419	0.606
No. of residues with additional alternate conformations in the X-ray model§		34	28	31	17
No. of re-refined waters§	X-ray	498	230	158	85
	Neutron	437	156	127	38
No. of common waters (within 0.5 Å)		364	131	81	16
Lone waters§ (percentage of total respective waters)	X-ray	134 (27%)	99 (43%)	77 (49%)	69 (81%)
	Neutron	73 (17%)	25 (16%)	46 (36%)	22 (58%)
Isotropic ADPs (Å^2^)					
Protein¶ (min/max/mean)	X-ray	5.0/73.8/9.8	8.3/54.5/16.5	8.9/47.5/17.9	7.7/41.6/15.7
	Neutron	1.2/107.8/7.8	21.2/88.7/36.0	2.7/131.8/13.8	6.0/53.3/15.3
Waters¶ (min/max/mean)	X-ray	4.9/56.3/27.8	10.1/60.3/36.9	11.2/68.3/35.2	13.3/67.9/34.7
	Neutron	1.0/50.4/22.6	24.6/56.5/41.9	5.3/28.1/15.5	8.3/45.7/23.9

† Reference and PDB metadata use a high-resolution limit of 1.3 Å.

‡ Recomputed based on deposited data (the completeness value in PDB entry metadata can often be ambiguous).

§ Passing the CC threshold.

¶ No CC threshold.

## Footnotes

1 The *X* in *X*—H denotes any heavy atom.
